# Genetically Raised Circulating Levels of Dietary Antioxidants and the Association With Respiratory Health in High‐Risk Populations

**DOI:** 10.1155/carj/5208730

**Published:** 2026-01-31

**Authors:** A. Saied, L. J. Horsfall

**Affiliations:** ^1^ University College London Research Department of Primary Care and Population Health, London, UK; ^2^ University College London Institute of Health Informatics, London, UK

**Keywords:** antioxidant, diet, lung function, Mendelian randomization

## Abstract

**Background:**

Observational studies of raised dietary antioxidants suggest a beneficial effect on respiratory health; however, findings from interventional trials have been inconsistent or null. Few studies have specifically targeted individuals exposed to high levels of environmental oxidants, where the antioxidant effects may be more pronounced.

**Objectives:**

To investigate whether genetically elevated serum levels of dietary antioxidants are causally associated with improved lung function and whether these effects differ with exposure to oxidative stress.

**Methods:**

We conducted a two‐sample Mendelian randomization (MR) study using summary‐level data for genetic associations with serum levels of ascorbic acid (vitamin C), retinol (vitamin A), and β‐carotene from published genome‐wide association studies. Outcome data on forced expiratory volume in one second (FEV_1_) and forced vital capacity (FVC) were derived from individual‐level data from over 285,000 UK Biobank participants. We used linear regression to estimate SNP‐outcome associations and applied the Wald ratio to derive causal estimates using published SNP‐exposure effect sizes.

**Results:**

We found no consistent evidence that genetically elevated serum antioxidant levels are associated with improved lung function. There was no evidence of effect modification by exposures linked to oxidative stress, including cigarette smoke, air pollution, or dietary factors.

**Conclusions:**

Our findings align with those of previous interventional studies, showing no consistent causal relationship between dietary antioxidants and respiratory health. Moreover, we found no strong support for targeted interventions to increase serum antioxidant levels in people exposed to high levels of environmental oxidants (Wellcome Grant ID: 209207/Z/17/Z and 225195/Z/22/Z).

## 1. Introduction

Maintaining healthy lung function is essential for overall well‐being and is a key determinant of respiratory and cardiovascular health [1–3]. Impaired lung function is associated with an increased risk of chronic respiratory diseases, such as asthma and chronic obstructive pulmonary disease (COPD), which together contribute substantially to the global burden of disease and premature mortality. For example, COPD alone is the fourth leading cause of death worldwide [4] and is projected to affect 600 million people globally by 2050 [5]. Lung function declines naturally with age; however, this process can be accelerated by environmental exposure and lifestyle factors. Therefore, identifying affordable and scalable strategies to preserve lung function across the course of life is an urgent public health priority.

Oxidative stress is an imbalance between oxidants (reactive oxygen and nitrogen species) and antioxidants that causes damage to tissues, proteins, and DNA. Reactive oxygen and nitrogen species within the lungs are mainly produced by phagocytes and polymorphonuclear, alveolar, bronchial, and endothelial cells. The lungs are continuously exposed to exogenous oxidants such as cigarette smoke, mineral dust, ozone, and radiation. Oxidative stress has been shown to have a role in the pathogenesis of diffuse lung diseases, and it has been hypothesized that increases in levels of circulating antioxidants can scavenge free radicals and protect against lung disease [6, 7].

Observational studies have linked higher dietary and serum antioxidant levels with improved health on a range of outcomes [8]. For example, serum levels of vitamins A, C, D, and α‐tocopherol vitamin E are associated with increased respiratory morbidity and mortality in a representative pooled analysis of 34,000 adults in the United States [9]. However, these associations are prone to confounding by other factors linked to a healthy lifestyle (e.g., physical activity) and reverse causation [10]. While randomized controlled trials (RCTs) offer stronger causal inference, findings have been inconsistent, and many trials suffer from limitations such as short duration, nontargeted populations, and supraphysiological dosing [11, 12]. There is also evidence that antioxidant supplementation may be more beneficial in individuals exposed to high levels of oxidative stress, such as smokers [13], yet few trials have tested this hypothesis directly.

Given the limitations of both observational and interventional evidence, Mendelian randomization (MR) offers an alternative approach to assess the causal effects of long‐term antioxidant exposure on lung function. MR uses genetic variants as instrumental variables to proxy lifelong differences in exposure, reducing bias from confounding and reverse causation under specific assumptions.

In this study, we apply a two‐sample MR design to investigate the causal effect of genetically predicted serum levels of ascorbic acid (vitamin C), retinol (vitamin A), and β‐carotene on lung function in adults from the UK Biobank. We hypothesize that higher genetically instrumented antioxidant levels are associated with better lung function and that these effects may be modified by environmental exposures such as smoking, diet, and air pollution.

## 2. Materials and Methods

### 2.1. Setting

UK Biobank is a cohort containing genotype data matched with deep phenotype data for 500,000 volunteer participants aged between 40 and 69, living in the UK [14]. Participants were recruited between April 2007 and August 2010 and provided data on their health using questionnaires, physical measurements by a trained nurse, and blood samples. Genome‐wide genotype data were available for the Affymetrix UK Biobank Axiom array (most participants) and the Applied Biosystems UK BiLEVE Axiom Array by Affymetrix (a smaller subset of *n* = 49,950). Details on the quality control and imputation of SNPs, indels, and structural variants are reported elsewhere [14].

### 2.2. Participants

The number of participants in the UK Biobank is 502,527. We excluded outliers for genotype missingness, excess heterozygosity, and sex discordance (*n* = 2200 excluded). We also removed close relatives to ensure that participants had no close family member in the study (*n* = 39,642 excluded). We further restricted the sample to “white British” participants using self‐reported ethnicity and the results of an existing principal components analysis (*n* = 88,341 excluded) [14]. Principal components capture ethnicity by summarizing patterns of genetic variation that reflect ancestral differences in allele frequencies across populations. Including these components as covariates in genetic analyses helps adjust for population structure and reduces confounding due to ethnicity. Online tools were used to confirm the adequacy of the sample size (http://cnsgenomics.com/shiny/mRnd/).

### 2.3. Selection of Genetic Variants

MR analysis relies on three core assumptions to support valid causal inference. First, the relevance assumption requires that the genetic variants used as instruments are robustly associated with the exposure of interest (in this case, circulating antioxidant levels). Second, the independence assumption stipulates that these genetic instruments are not associated with confounders of the exposure–outcome relationship. Third, the exclusion restriction assumption requires that genetic variants influence the outcome (lung function) solely through their effect on the exposure and not via alternative biological pathways (i.e., no horizontal pleiotropy).

We identified genetic variants as instrumental variables for circulating concentrations of ascorbic acid (vitamin C), retinol (vitamin A), and β‐carotene. Because the UK Biobank does not contain direct biomarker measurements for these antioxidants, we applied a two‐sample MR strategy. SNP‐exposure associations were taken from external summary‐level GWAS datasets, and SNP‐outcome associations were derived from individual‐level UK Biobank data.

To identify suitable instruments, we searched the NHGRI‐EBI GWAS Catalog (http://www.ebi.ac.uk/gwas/) and conducted a broader literature review using PubMed. We selected independent SNPs most strongly associated with serum antioxidant levels, prioritizing studies conducted in predominantly European ancestry populations to match the UK Biobank outcome dataset. Specifically, we included a candidate gene analysis for ascorbate and genome‐wide association studies (GWASs) for circulating β‐carotene and retinol.

The SNP used to instrument ascorbate is a missense variant in *SLC23A1*, which encodes the sodium‐dependent vitamin C transporter 1 (SVCT1), a key protein in intestinal absorption and active transport of dietary vitamin C [15]. The SNPs for β‐carotene are located in *BCO1* (also known as *BCMO1*), which encodes beta‐carotene oxygenase 1, an enzyme that cleaves carotenoids into retinal in the small intestine [16]. The SNP instrumenting retinol is located near *RBP4*, which encodes retinol‐binding protein 4, responsible for transporting retinol from liver stores to peripheral tissues [17]. Mutations in *RBP4* have been linked to vitamin A deficiency and related disorders, such as retinitis pigmentosa [18]. Further details on the summary statistics used in the statistical analyses are included in Supporting Table S1.

### 2.4. Outcomes

The primary outcome is lung function, measured as forced expiratory volume in one second (FEV_1_) and forced vital capacity (FVC). Breath spirometry was performed on participants attending the assessment center using the Vitalograph Pneumotrac 6800. The participant was asked to record two to three blows (lasting for at least 6 s) within approximately 6 min. The computer then compared the reproducibility of the first two blows and, if acceptable (defined as *a* < 5% difference in FVC and FEV_1_), indicated that the third blow was not required. Our previous work found that introducing these types of quality control to lung function can exclude people with the lowest lung function and may introduce selection bias. Therefore, we also analyzed the best blow recorded without exclusions based on acceptability as a sensitivity analysis.

### 2.5. Covariates

To improve the precision of our estimates, we included known predictors of variation in lung function in our models. The variables included age, genetic sex, height, weight, and self‐reported smoking status. We also included the recruitment center and the first 40 principal components provided by the UK Biobank [14] to help adjust for any population substructure within the “white British” participants. We tested for interactions with variables that might influence the levels of oxidative stress, including smoking status (categorical variable: never, former, and current), pack‐years of smoking (continuous variable), daily dietary supplements containing purported antioxidants (binary), and area‐linked levels of NO_2_ air pollution (continuous).

### 2.6. Assumptions

MR analysis relies on three core assumptions to support valid causal inference. First, the relevance assumption requires that the genetic variants used as instruments are robustly associated with the exposure of interest, in this case, the circulating antioxidant levels. Second, the independence assumption stipulates that these genetic instruments are not associated with confounders of the exposure–outcome relationship. Third, the exclusion restriction assumption requires that the genetic variants influence the outcome (lung function) solely through their effect on the exposure and not via alternative biological pathways (i.e., no horizontal pleiotropy). To strengthen these assumptions, we selected biologically plausible and well‐characterized genetic variants located in genes directly involved in antioxidant transport and metabolism. We also examined the associations between the selected genotypes and key covariates to assess potential violations of the independence assumption. As each antioxidant was instrumented using a single genetic variant, we did not perform formal tests for horizontal pleiotropy (e.g., MR‐Egger), which requires multiple instruments.

### 2.7. Statistical Analyses

SNP alleles were coded additively as 0, 1, or 2, representing the number of effect alleles associated with increased circulating levels of the respective antioxidants. To assess the assumption of linearity in the genotype–phenotype relationship, we also modeled SNPs as categorical variables in sensitivity analyses. All continuous variables, including lung function measures, height, weight, pack‐years of smoking, fruit and vegetable intake, and NO_2_ exposure, were retained in their continuous form to preserve the statistical power and avoid arbitrary categorization.

We initially investigated the cross‐sectional relationships between SNPs associated with circulating antioxidant levels and lung function. We then instrumented the strength of the association using published summary statistics (beta coefficients) for serum antioxidants and individual‐level lung function data measured in UK Biobank participants at baseline (two‐sample MR). We initially identified and excluded outlier continuous values using a multivariate approach (blocked adaptive computationally efficient outlier nominators algorithm) with a 15% threshold of the chi‐squared distribution to separate outliers from nonoutliers. We used linear regression to estimate the association between genotypes and lung function after including important predictors (e.g., sex at birth) and potential confounders (e.g., principal components). To visualize potential gene–environment interactions, we calculated the margins of response as adjusted FEV_1_ and FVC values in liters for different levels of environmental exposure linked to oxidative stress (smoking, air pollution, and poor diet) while holding all other variables at their observed values. We used the Wald test to calculate the *p*‐values associated with the interaction terms. To estimate the causal effect of a unit increase in each circulating antioxidant on lung function, we extracted beta coefficients of the SNP exposure from the literature and combined these with the beta coefficients for SNP outcome from the UK Biobank using the Wald ratio [19]. The Wald ratio was calculated by dividing the genetic association with the outcome (FEV_1_) by the genetic association with the exposure (antioxidant level), providing an estimate of the causal effect per standard‐unit increase in antioxidant concentration. This ratio reflects the expected change in lung function attributable to a genetically predicted increase in serum antioxidant levels, assuming that the instrumental variable assumptions hold. As the exposure was from summary data, it was not possible to use the same covariate adjustment strategy for SNP‐exposure/SNP‐outcome associations.

We performed several sensitivity analyses to test the robustness of our findings. Lung function data were missing for approximately 25% of participants, and missingness was associated with several other risk factors, such as smoking status; therefore, the data were not missing at random. As a sensitivity analysis, we imputed missing lung function and other continuous variables (height and weight) using multivariate normal regression and recalculated the associations [20]. This method uses an iterative Markov chain Monte Carlo method to impute missing values, and we used *n* = 10 imputations for this. The recent use of respiratory medications was self‐reported at baseline. These medications could affect spirometry readings; therefore, we also assessed the impact of excluding participants who reported taking these drugs. Statistical analyses were performed using Stata Version 16.1 and R Version 4.2.2.

This manuscript was written using the Strengthening the Reporting of Observational Studies in Epidemiology Using Mendelian Randomization (STROBE‐MR) Statement [21]. A shorter version of this work was presented as a poster at the European Society for Human Genetics [22] and published as an abstract, with a full version published as a preprint [23]. The present manuscript is longer and contains a detailed literature review, extensive methodology, additional results, in‐depth analyses, and a thorough discussion of the results. The use of any Artificial Intelligence Generated Content (AIGC) tools such as ChatGPT and others based on large language models (LLMs) has not been used in developing any portion of the manuscript. A shorter version of this work was presented as a poster at the European Society for Human Genetics [22] and published as an abstract and a full version published as a preprint [23]. The present manuscript is longer and contains a detailed literature review, extensive methodology, additional results, in‐depth analyses, and a thorough discussion of results.

UK Biobank has been approved by the North West Multicenter Research Ethics Committee as a research tissue bank. The present study protocol was preregistered and approved by the UK Biobank Access Committee (ID: 5167).

## 3. Results

Approximately 285,000 participants had data on both the genetic variants of interest and lung function (Table 1). The mean FEV_1_ was 2.85 (SD ± 0.77), and the mean FVC was 3.78 (SD ± 0.97). There was no clear association between the alleles associated with increased retinol or β‐carotene levels and increased lung function (Table 1). In participants with alleles associated with increased ascorbate, FEV_1_ was 9 mL higher and FVC was 10 mL higher. The unadjusted and adjusted coefficients showed a positive association between alleles associated with increased circulating antioxidants and lung function in most analyses (Table 2). The positive association between alleles that increase ascorbate levels and lung function was reversed after adjustment. For example, before adjustment, each additional effect allele was associated with a 11.7 mL increase in FEV_1_ (95% CI: 1.6–21.9), which was reduced to 1.3 mL (−6.0–8.6) after adjustment. Further analyses found that a slight imbalance in sex and height across ascorbate genotypes explained the positive relationship suggested by Table 1 and the unadjusted analyses in Table 2. Imputing missing data for continuous variables substantially increased our analytic sample size but did not alter our findings (Table 2). We found no clear evidence of nonadditive effects or interactions between the genotypes and smoking status (Table 3).

**TABLE 1:** Characteristics of UK Biobank participants included in the analysis overall and by number of effect alleles.

**Number of effect alleles**						

**rs4889286**	**Total**	**0 effect alleles**	**1 effect allele**	**2 effect alleles**	***n***	**Missingness**

(β‐carotene)	*N* = 374,924	*N* = 82,766	*N* = 186,850	*N* = 105,308	374,924	
Male sex	173,644 (46.3%)	38,237 (46.2%)	86,560 (46.3%)	48,847 (46.4%)	374,924	0%
Age at recruitment^∗^	58.9 (51.4–64.0)	58.9 (51.3–64.0)	58.9 (51.4–63.9)	58.9 (51.3–64.0)	374,924	0%
Weight (kg)	78.3 (15.5)	78.3 (15.5)	78.3 (15.4)	78.2 (15.4)	373,875	0%
Height (cm)	168.8 (9.1)	168.8 (9.2)	168.8 (9.1)	168.8 (9.1)	374,166	0.2%
Smoking status					374,924	0%
Never	204,092 (54.4%)	45,048 (54.4%)	101,851 (54.5%)	57,193 (54.3%)		
Former	131,731 (35.1%)	29,141 (35.2%)	65,502 (35.1%)	37,088 (35.2%)		
Current	37,822 (10.1%)	8314 (10.0%)	18,849 (10.1%)	10,659 (10.1%)		
Missing	1279 (0.3%)	263 (0.3%)	648 (0.3%)	368 (0.3%)		
Pack‐years of smoking	19.5 (10.0–32.5)	19.5 (10.0–32.5)	19.5 (10.0–32.5)	19.5 (10.1–32.4)	114,350	32% (of ever smokers)
Use of antioxidant supplements	101,807 (27.2%)	22,591 (27.3%)	50,682 (27.1%)	28,534 (27.1%)	374,924	0%
Nitrogen dioxide air pollution; 2010 (μg/m^3^)	25.6 (21.0–30.4)	25.5 (21.0–30.3)	25.5 (20.9–30.4)	25.6 (21.0–30.4)	369,917	2%
FEV_1_	2.85 (0.77)	2.85 (0.77)	2.85 (0.76)	2.85 (0.77)	284,486	24%
FVC	3.78 (0.97)	3.78 (0.97)	3.78 (0.97)	3.78 (0.97)	284,486	24%

**rs10882272**	**Total**	**0 effect alleles**	**1 effect alleles**	**2 effect alleles**	***n***	**Missingness**

Retinol	*N* = 376,771	*N* = 54,228	*N* = 176,918	*N* = 145,625	376,771	
Male sex	174,538 (46.3%)	24,937 (46.0%)	82,392 (46.6%)	67,209 (46.2%)	376,771	0%
Age at recruitment∗	58.9 (51.3–64.0)	58.9 (51.3–64.0)	58.9 (51.4–63.9)	58.9 (51.3–64.0)	376,771	0%
Weight (kg)	78.3 (15.5)	78.1 (15.4)	78.3 (15.5)	78.3 (15.5)	375,715	0%
Height (cm)	168.8 (9.1)	168.7 (9.1)	168.9 (9.1)	168.8 (9.2)	376,010	0.2%
Smoking status					376,771	0%
Never	205,088 (54.4%)	29,460 (54.3%)	96,403 (54.5%)	79,225 (54.4%)		
Former	132,373 (35.1%)	19,030 (35.1%)	62,073 (35.1%)	51,270 (35.2%)		
Current	38,022 (10.1%)	5562 (10.3%)	17,842 (10.1%)	14,618 (10.0%)		
Missing	1288 (0.3%)	176 (0.3%)	600 (0.3%)	512 (0.4%)		
Pack‐years of smoking	19.5 (10.0–32.5)	19.5 (10.0–32.5)	19.5 (10.0–32.5)	19.5 (10.1–32.4)	114,350	32% (of ever smokers)
Use of antioxidant supplements	101,807 (27.2%)	22,591 (27.3%)	50,682 (27.1%)	28,534 (27.1%)	374,924	0%
Nitrogen dioxide air pollution; 2010 (μg/m^3^)	25.6 (21.0–30.4)	25.5 (21.0–30.3)	25.5 (20.9–30.4)	25.6 (21.0–30.4)	369,917	2%
FEV_1_	2.85 (0.77)	2.85 (0.77)	2.86 (0.77)	2.85 (0.76)	285,877	24%
FVC	3.78 (0.97)	3.77 (0.97)	3.79 (0.97)	3.78 (0.97)	285,877	24%

**rs33972313**	**Total**	**0 effect alleles**	**1 effect alleles**	**2 effect alleles**	***n***	**Missingness**

Ascorbate	*N* = 376,771	*N* = 459	*N* = 25,454	*N* = 350,858	376,771	
Male sex	174,538 (46.3%)	188 (41.0%)	11,716 (46.0%)	162,634 (46.4%)	376,771	0.00%
Age at recruitment∗	58.9 (51.3–64.0)	57.6 (49.8–62.8)	58.9 (51.2–64.0)	58.9 (51.4–64.0)	376,771	0.00%
Weight (kg)	78.3 (15.5)	77.8 (15.5)	78.2 (15.5)	78.3 (15.4)	375,715	0.28%
Height (cm)	168.8 (9.1)	167.9 (8.7)	168.7 (9.1)	168.8 (9.2)	376,010	0.20%
Smoking status					376,771	0.00%
Never	205,088 (54.4%)	239 (52.1%)	13,827 (54.3%)	191,022 (54.4%)		
Former	132,373 (35.1%)	166 (36.2%)	8989 (35.3%)	123,218 (35.1%)		
Current	38,022 (10.1%)	51 (11.1%)	2562 (10.1%)	35,409 (10.1%)		
Missing	1288 (0.3%)	3 (0.7%)	76 (0.3%)	1209 (0.3%)		
FEV_1_	2.85 (0.77)	2.76 (0.76)	2.85 (0.76)	2.85 (0.77)	285,877	24.12%
FVC	3.78 (0.97)	3.68 (0.94)	3.78 (0.96)	3.78 (0.97)	285,877	24.12%

*Note:* All continuous variables are means and standard deviations except where indicated.

^∗^Median with interquartile range.

**TABLE 2:** Genetic variants influencing circulating dietary antioxidants and their association with lung function in UK Biobank participants.

		**FEV**_**1**_ **(mL)**	**FVC (mL)**	***N***

Best measure of attempts assessed as acceptable	Unadjusted per allele effect (95% CI)			
	rs4889286 (β‐carotene)	1.1 (−3.0–5.1)	1.7 (−3.5–6.8)	284,717
	rs10882272 (Retinol)	1.6 (−2.6–5.7)	2.3 (−3.0–7.5)	286,109
	rs33972313 (Ascorbate)	5.6 (−5.4–16.6)	8.4 (−5.5–22.3)	286,109
	Adjusted per allele effect^∗^ (95% CI)			
	rs4889286	0.9 (−2.0–3.9)	1.3 (−2.0–4.7)	284,498
	rs10882272	0.8 (−2.2–3.8)	1.4 (−2.1–4.8)	285,888
	rs33972313	−2.2 (−10.2 to 5.7)	−2.7 (−11.7 to 6.4)	285,888

Best of all measurements	Unadjusted per allele effect (95%CI)			
	rs4889286	1.4 (−2.3–5.2)	0.8 (−4.3–5.9)	342,522
	rs10882272	1.6 (−2.3–5.4)	2.6 (−2.6–7.9)	344,202
	rs33972313	11.7 (1.6–21.9)	18.8 (4.9–32.6)	344,202
	Adjusted per allele effect^∗^ (95% CI)			
	rs4889286	1.4 (−1.3–4.1)	0.6 (−3.0–4.3)	341,893
	rs10882272	0.3 (−2.5–3.1)	1.2 (−2.6–5.0)	343,568
	rs33972313	1.3 (−6.0–8.6)	4.1 (−5.8–14.1)	343,568

Multiple imputation	Unadjusted per allele effect (95% CI)			
	rs4889286	1.7 (−2.0–5.3)	1.1 (−3.9–6.1)	375,433
	rs10882272	1.4 (−2.3–5.2)	2.7 (−2.4–7.8)	377,115
	rs33972313	12.1 (2–22.1)	18.6 (5–32.2)	377,115
	Adjusted per allele effect^∗^ (95% CI)			
	rs4889286	1.6 (−1.1–4.3)	0.8 (−2.8–4.5)	375,433
	rs10882272	0.1 (−2.6–2.9)	1.1 (−2.6–4.8)	377,115
	rs33972313	1.3 (−6.2–8.7)	3.7 (−6.3–13.7)	377,115

^∗^Adjusted for sex, age, height, weight, and smoking status.

**TABLE 3:** Interaction between smoking status and genetic variants influencing circulating dietary antioxidants and their association with lung function.

Smoking status	No. effect alleles	rs4889286 (β‐carotene)	rs10882272 (retinol)	rs33972313 (ascorbate)
*Predicted* *F* *E* *V*_1_ in liters (95% CI)*^∗^*				
Never	0	2.92 (2.91–2.92)	2.91 (2.91–2.92)	2.87 (2.79–2.95)
	1	2.92 (2.91–2.92)	2.92 (2.91–2.92)	2.92 (2.91–2.93)
	2	2.92 (2.91–2.92)	2.92 (2.91–2.92)	2.92 (2.91–2.92)

Former	0	2.80 (2.79–2.8)	2.80 (2.79–2.81)	2.77 (2.67–2.86)
	1	2.80 (2.80–2.81)	2.80 (2.80–2.81)	2.80 (2.79–2.82)
	2	2.80 (2.80–2.81)	2.80 (2.79–2.81)	2.80 (2.80–2.80)

Current	0	2.69 (2.68–2.71)	2.70 (2.68–2.72)	2.83 (2.65–3.02)
	1	2.70 (2.69–2.71)	2.70 (2.69–2.71)	2.71 (2.68–2.73)
	2	2.71 (2.7–2.72)	2.71 (2.70–2.72)	2.70 (2.69–2.71)

*P* for interaction		0.29	0.24	0.34

Predicted FVC in liters (95% CI)*^∗^*				
Never	0	3.83 (3.82–3.83)	3.82 (3.82–3.83)	3.77 (3.68–3.86)
	1	3.83 (3.82–3.83)	3.83 (3.82–3.83)	3.83 (3.82–3.84)
	2	3.83 (3.82–3.83)	3.83 (3.82–3.83)	3.83 (3.82–3.83)

Former	0	3.74 (3.73–3.74)	3.74 (3.73–3.75)	3.74 (3.63–3.84)
	1	3.74 (3.74–3.75)	3.74 (3.74–3.75)	3.74 (3.73–3.76)
	2	3.74 (3.73–3.75)	3.74 (3.73–3.74)	3.74 (3.74–3.74)

Current	0	3.70 (3.69–3.72)	3.71 (3.69–3.72)	3.91 (3.71–4.11)
	1	3.71 (3.70–3.72)	3.71 (3.70–3.72)	3.72 (3.69–3.75)
	2	3.72 (3.71–3.73)	3.72 (3.70–3.73)	3.71 (3.70–3.72)

*P* for interaction		0.5	0.27	0.12

^∗^Adjusted for sex, height, weight, ethnicity principal components, smoking, and recruitment center.

**FIGURE 1:**
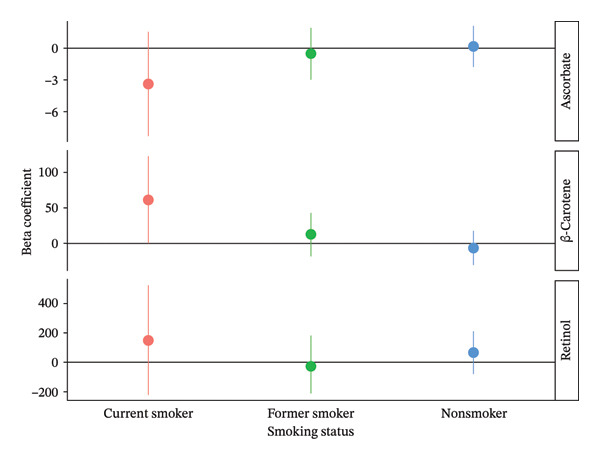
MR results for antioxidant exposure and lung function according to self‐reported smoking status. β coefficients and 95% confidence intervals show the change in FEV_1_ (mL) per μmol/L for ascorbate and log μg/L for β‐carotene and retinol.

The Wald ratio was used to estimate the causal effects of a standard‐unit increase in antioxidant levels on lung function (Figure 1). In current smokers, higher β‐carotene levels were positively associated with FEV_1_, suggesting a potential protective effect, although the confidence intervals were wide. Retinol also showed a large positive beta in smokers but with substantial uncertainty. In contrast, ascorbate was negatively associated with FEV_1_ in current smokers, although the confidence interval crossed zero. No consistent associations were observed in former or nonsmokers.

Excluding people on lung medication did not meaningfully change the results, and there was no evidence of interactions with pack‐years of smoking, air pollution levels, or use of vitamin supplements (data not shown).

## 4. Discussion

### 4.1. Summary

This study examined whether genetically raised circulating dietary antioxidants are associated with improved lung function. We found no convincing evidence of protective effects in ex‐ or never‐smokers. Although the positive association between β‐carotene and FEV_1_ in current smokers may suggest a potential protective effect, the wide confidence intervals and lack of consistent associations across smoking categories raise concerns regarding the robustness of the estimates. The large but imprecise effect observed for retinol and the negative association for ascorbate with overlapping confidence intervals further underscore this uncertainty. This suggests that long‐term exposure to slightly elevated serum ascorbate (vitamin C), retinol (vitamin A), and β‐carotene is unlikely to protect lung health in a clinically meaningful way, even in people exposed to high levels of oxidants. However, some uncertainty remains regarding the potential effects in current smokers, and larger, well‐powered studies are needed to confirm whether protective associations exist in this subgroup.

### 4.2. Strengths and Limitations

The main strength of this study was the use of genetic instruments to reduce bias from reverse causation and residual confounding that can be present in studies using measured blood antioxidant levels. Other strengths include the large sample size and the ability to examine subsets of participants exposed to higher levels of oxidants, which is not usually possible with MR analyses using summary‐level data.

Limitations include the self‐reporting of smoking status and other variables used to estimate exposure to oxidative stress. Exposure misclassification and poor measurement may have reduced our ability to detect important interactions between variables. We lacked the statistical power to examine interactions with clinical endpoints, such as lung cancer diagnosis or mortality. It is recognized that there is selection bias among people who volunteer to join the UK Biobank, with smokers and people with known respiratory diseases being significantly underrepresented [24, 25]. A true causal association might be diluted if people with genetically low antioxidants are less likely to participate in the UK Biobank because of death or poor health [26]. Furthermore, because the GWAS studies were based on white Europeans, we restricted our analysis to white British participants. This limitation affects the generalizability of our findings. When the instruments were selected for this study, we identified only one unlinked, biologically plausible SNP for each antioxidant of interest. This restricts the power of our analyses and inhibits our ability to perform additional checks for evidence of bias due to the horizontal pleiotropy. As more GWAS studies become available on circulating dietary antioxidants, the instruments can be expanded, and the precision in estimating causal effects can be increased.

Pleiotropic effects may also violate the third condition of an instrumental variable. The instrument for raised serum vitamin C, the SNP at location rs33972313, was weakly associated with height. On average, participants with effect alleles were almost 1 cm taller (167.9 cm vs. 168.8 cm). Two otherwise identical participants at these heights would have a 1.5% difference in their FVC measurements. It seems biologically plausible that someone with higher levels of dietary‐derived nutrients in their blood might be taller, anatomically increasing lung function, independently of an antioxidant mechanism. It is also plausible that our selected SNPs influence the levels of other antioxidants or metabolites. For example, there is some evidence that SNPs that increase serum levels of β‐carotene decrease serum levels of other carotenoids [16]. The risk of bias from sample overlap in each exposure and outcome analysis cannot be excluded, but it is likely to be low due to differences in geographic location and the time period of data collection.

### 4.3. Comparison With Other Studies

The role of antioxidants in health outcomes has been extensively studied, with evidence linking them to reduced morbidities and mortalities. A prospective cohort study reported an inverse relationship between plasma ascorbic acid levels and all‐cause mortality, suggesting the potential of vitamin C to lower mortality risk [27]. Another study found that higher vitamin E consumption was associated with a reduced risk of coronary heart disease in men [28]. These studies were large and used robust exposure assessments, although residual confounding remains a major limitation. Research has shown positive associations between antioxidant intake and respiratory function [29, 30]. One large cross‐sectional analysis (MORGEN study) linked lower antioxidant consumption to increased respiratory symptoms, and another supported these findings with data from a national survey (NHANES III) [30]. Both studies were limited by their reliance on self‐reported dietary data and residual confounding. Further evidence from a systematic review suggests that antioxidant supplementation may mitigate pollutant‐induced declines in lung function [31], while a pooled analysis demonstrated reduced respiratory morbidity and mortality associated with higher serum antioxidant levels [31].

Despite observational studies suggesting the benefits of antioxidants, RCTs have often failed to establish causality. A large RCT investigating vitamin E and beta‐carotene supplementation in male smokers found no reduction in lung cancer incidence and even observed potential harm with beta‐carotene [32]. Another trial examining vitamin E and selenium supplementation showed no significant impact on lung function decline, further challenging the hypothesis of antioxidant efficacy in respiratory health [13]. Similarly, the Heart Protection Study, which involved over 20,000 high‐risk individuals, found no cardiovascular or cancer benefits from antioxidant supplementation [33]. Furthermore, meta‐analyses of vitamin C supplementation in COPD and preventive trials for cardiovascular disease and cancer have provided limited evidence of efficacy [11, 12]. The discrepancies between observational and trial evidence are attributed to confounding, reverse causation, and publication bias in observational studies [8]. Residual confounding is particularly pervasive, as people with diets high in antioxidants generally have higher levels of income, physical activity, and many other exposures that could explain the observed relationships. Another important bias is reverse causation, in which people possibly change their antioxidant intake after becoming unwell. Additionally, lung damage may affect the utilization of circulating antioxidants.

MR studies offer some advantages over RCTs, including the ability to assess lifelong exposure to lower, physiologically relevant doses of nutrients that better reflect typical dietary intake. These studies have yielded conflicting results. For example, an MR study involving over 1 million participants found no causal link between diet‐derived antioxidants and ischemic stroke [34]. Similarly, another MR analysis found no significant protective effect of circulating antioxidants on coronary heart disease risk [35]. Conversely, a recent MR study indicated the potential benefits of selenium and retinol in reducing stroke risk, although these results were tenuous and require further replication [36]. These results underscore the complexity of antioxidant research, with MR studies generally emphasizing that previously observed associations may not be causal. While MR provides evidence against certain claims, it also highlights the need to explore subpopulations or conditions in which antioxidants may exert clinically meaningful effects. This was the focus of the present study, where access to individual‐level data enabled subgroup analyses of high‐risk groups that are not always possible with summary data frequently used in MR.

### 4.4. Future Directions

Understanding the causal relationship between dietary antioxidants is a vital area of research, given the low cost of intervention and the high burden of oxidative stress‐mediated diseases. Future large‐scale MR studies with multiple genetic instruments and repeated lung function measurements for participants recruited at a younger age to reduce selection bias will improve the causal evidence base for dietary antioxidants. Well‐designed RCTs investigating the effects of recommended daily amounts of antioxidant vitamins on lung function in young people living in areas with high concentrations of airborne oxidants would also be a valuable addition to the evidence base.

## 5. Conclusion

We found no clear relationship between genetic variants influencing serum dietary antioxidant levels and lung function, overall, or in individuals exposed to higher levels of oxidative stress. In conclusion, this study, together with the existing evidence base, raises questions about the role of dietary antioxidants as modifiable factors for improved lung health, even in high‐risk populations such as smokers. However, some uncertainty remains, particularly in current smokers, and further large‐scale studies are needed to clarify whether protective effects exist in this subgroup.

## Author Contributions

Conceptualization: A. Saied and L. J. Horsfall; methodology: A. Saied and L. J. Horsfall; software: A. Saied and L. J. Horsfall; formal analysis: L. J. Horsfall; investigation: A. Saied and L. J. Horsfall; resources: L. J. Horsfall; data curation: L. J. Horsfall; writing–original draft preparation: A. Saied; writing–review and editing: L. J. Horsfall; visualization: A. Saied; supervision: L. J. Horsfall; project administration: L. J. Horsfall; funding acquisition: L. J. Horsfall.

## Funding

This research was funded in whole, or in part, by the Wellcome Trust (Grant number 209207/Z/17/Z and 225195/Z/22/Z). For the purpose of open access, the author has applied a CC BY public copyright license to any Author Accepted Manuscript version arising from this submission.

## Disclosure

All authors have read and agreed to the published version of the manuscript. A preprint of this work has been previously published [1]. The funders had no role in the study’s design; in the collection, analyses, or interpretation of data; in the writing of the manuscript; or in the decision to publish the results.

## Ethics Statement

UK Biobank has ongoing approval from the North West Multi‐center Research Ethics Committee as a Research Tissue Bank (RTB) approval. This approval means that researchers do not require separate ethical clearance and can carry out research under the RTB approval. The present study protocol has been approved by the UK Biobank (ID: 5167).

## Conflicts of Interest

The authors declare no conflicts of interest.

## Supporting Information

The supporting information provides additional details supporting the genetic instruments used in this study. Supporting Table S1 summarizes the single nucleotide polymorphisms (SNPs) selected for each exposure following harmonization, including the associated genes, effect and noneffect alleles, effect sizes (beta), standard errors, *p*‐values, units of measurement, and effect allele frequencies. The table also reports summary statistics for circulating levels of ascorbate, β‐carotene, and retinol from the original GWASs, along with references to the primary data sources. These details allow transparent assessment of instrument selection and strength and provide essential context for the MR analyses presented in the main manuscript (Supporting Table S1).

## Supporting information


**Supporting Information** Additional supporting information can be found online in the Supporting Information section.

## Data Availability

The data supporting this study’s findings are available from UK Biobank (https://www.ukbiobank.ac.uk/enable-your-research/apply-for-access). Restrictions apply to the availability of these data under Material Transfer Agreements.
