# Supplementary material for: Genetically Raised Circulating Levels of Dietary Antioxidants and the Association With Respiratory Health in High‐Risk Populations
Source: Can Respir J. 2026 Jan 31;2026:5208730. doi: 10.1155/carj/5208730 (PMC12860137; doi:10.1155/carj/5208730)
Supplement: Supplementary file 1 — Supporting Information Additional supporting information can be found online in the Supporting Information section. [file CARJ-2026-5208730-s001.docx]

Table S1: Information extracted from published studies on the SNPs used in each analysis after harmonisation

| **SNP** | **Exposure** | **Gene** | **Effect allele** | **Other allele** | **Beta** | **SE** | **P-val** | **Units** | **Effect allele frequency** | **Mean/median level** |  |
| --- | --- | --- | --- | --- | --- | --- | --- | --- | --- | --- | --- |
| **rs33972313*** | Ascorbate | SLC23A1 | T | C | -5.98 | 1.15 | 9.5E-08 | umol/l | 0.04 | Median (IQR) for BWHHS: 40 μmol/L (21-60 μmol/L). | DOI: 10.3945/ajcn.2010.29438 |
| **rs4889286**** | B-carotene | BCO1 | T | C | 0.1762 | 0.022 | 5.8E-16 | logn-ug/l | 0.5 | Mean (SD) for InCHIANTI: 0.41 (0.27) | DOI: 10.1016/j.ajhg.2008.12.019 |
| **rs10882272***** | Retinol | RBP4 | C | T | -0.03 | 0.004 | 7.0E-15 | logn-ug/l | 0.35 | Not reported overall. 579.7 μg/l for effect alleles = 0. No measure of variability.. | DOI: 10.1093/hmg/ddr387 |

* Data sources used: Discovery sample: BWHHS. Replication: EPIC-Norfolk, MIDSPAN, Family gen. 2, Ten Towns, BRHS

** Data sources used: InCHIANTI, WHAS I, WHAS II, ATBC

*** Data sources used: Discovery sample: ATBC, PLCO. Replication: InCHIANTI
